# Malawian mothers’ experiences of raising children living with albinism: A qualitative descriptive study

**DOI:** 10.4102/ajod.v10i0.693

**Published:** 2021-04-20

**Authors:** Naomi Likumbo, Tania de Villiers, Una Kyriacos

**Affiliations:** 1 Division of Nursing and Midwifery, Department of Health and Rehabilitation Sciences, Faculty of Health Sciences, University of Cape Town, Cape Town, South Africa

**Keywords:** albinism, albinism in Africa, albinism in Malawi, albinism killings, children living with albinism, maternal experiences, oculocutaneous albinism, stigma and albinism

## Abstract

**Background:**

Albinism in humans is characterised by a reduced amount of pigment (melanin) present in the skin, hair follicles and the eye; approximately 7000–10 000 Malawians of all ages are affected. Children with these features face extreme forms of human rights abuses, even death.

**Objectives:**

This study aims to describe Malawian mothers’ experiences, perceptions and understanding of raising children with albinism (CWA).

**Methods:**

The study was conducted in 2018 using a qualitative descriptive design, with purposive sampling and voluntary participation. Mothers, 18 years and older, who had given birth to a CWA and who attended the dermatology clinic of a local public hospital participated. An interview guide used during standardised, open-ended interviews was translated from English to Chichewa using forward and backward translation. Interviews were conducted in Chichewa, audio recorded, transcribed and forward and back translated from English to Chichewa. Thematic data analysis was employed.

**Results:**

The mean age of participants (*N* = 10) was 33 years; two had albinism. Emerging themes confirmed the existence of myths and stereotypes regarding albinism but from the mothers’ perspectives. Mothers reported: (1) some experiences of emotional pain, initially, but also love and acceptance of their children, despite adverse reactions of others; (2) their experiences of stigmatisation of their children and themselves, and of intended harm to their children, and (3) their own lack of knowledge and understanding of albinism.

**Conclusion:**

In our limited study, mothers’ self-reported experiences of raising CWA in Malawi highlight the need for educational programmes on albinism at national level, particularly for families with a CWA, health professionals and educators.

## Introduction

‘Albinism represents a group of inherited abnormalities of melanin synthesis’ (Oetting, Brilliant & King 1996:330) in the skin, hair follicles and parts of the eye responsible for vision. More melanin, resulting in darker skin colour, protects individuals from the harmful effects of ultraviolet (UV) light. People with albinism have a reduced amount of melanin or no melanin at all. In the group of conditions associated with albinism, only oculocutaneous albinism (OCA) is relevant to the present study. Oculocutaneous albinism is characterised by hypo-pigmentation or lack of pigmentation of the iris and retina of the eyes, hair follicles and skin (Kromberg 2018a) when compared with others of the same ethnic and racial backgrounds. Individuals who have OCA have pathogenic variants in both copies of the relevant OCA gene and inherit one of these from each parent, both of whom are carriers (National Health Service 2017). For persons with OCA, exposure to intense sunlight puts them at risk of developing skin cancer, which can result in morbidity and mortality (Kromberg 2018a) but in itself, albinism does not cause mortality (Tandon 2016). Although data are not current, a prevalence of albinism of 1/1000 is reported for a few specific ethnic group isolates in southern Africa, and an overall estimate ranging from 1/5000–1/15 000 has been made for the general population (Hong, Zeeb & Repacholi 2006). As albinism affects individuals and their families medically, socially and psychologically, access to healthcare and to a range of healthcare and disability practitioners is essential.

The wide-ranging effects of albinism are associated with disability (WHO 2018b). In the context of albinism, disability is defined in terms of activity limitations (because of visual impairment which may require assistive devices such as low vision aids) and participation restrictions (in outdoor activities because of extreme sensitivity of the skin to UV radiation (WHO 2018a). The WHO’s overview of disability (2018b) confirms that presently the understanding of disability is not confined to a physical or medical perspective, but takes into consideration a person’s physical, social and political context.

The medical model and definition of disability may be more commonly understood amongst healthcare practitioners (HCPs) than viewing disability as a social constructionist paradigm (Donoghue 2003). Over time, the traditional medical approach to disability has been replaced by three types of inquiries into disability: the ontological, epistemological and the experiential (Oliver 1996). Persons living with albinism (PLWA) primarily are social beings whose bodies may be afflicted with a medical disorder that makes them physically different from others, but who still need social support, a basic human need (Chang & Schaller 2000). In the context of African American ancestry and the association with hypertension, ‘[T]he hypothesis that skin colour is a marker of exposure to social stressors refers to the *cultural significance of skin colour* as a criterion of social classification’ (Gravlee, Dressler & Bernard 2005:2191). The social and emotional impact of a skin condition can be considerable and can lead to social inequity (Papadopoulos, Bor & Legg 1999), which might have implications for PLWA.

A person with poor vision who requires optical correction and may need special equipment to be employable, and who cannot work in a sun-exposed environment without costly protective clothing or a topical sunscreen, is often stigmatised (Cruz-Inigo, Ladizinski & Sethi 2011). If one’s physical features are very different from the rest of society, this can lead to discrimination and possibly preclude employment which can result in poverty (Gravlee et al. 2005). When considering albinism, the concept of disability, in its broadest sense (Oliver 1996), is more applicable and more useful than applying the medical model.

Parental guilt is sometimes associated with genetic conditions. In a South African study, guilt was less evident amongst parents with formal tertiary education (16/174, 6.3%) and amongst parents of children with visual impairment (CWVI) (14.3%) (Useh 2008). Of the parents of CWVI which included albinism, 82.1% displayed grief, 75% anger and 67.9% attributed their guilt to the double impairment of skin and sight and to societal prejudices and stigma associated with albinism (Useh 2008).

Socio-demographic statistics indicate that landlocked Malawi, located in Southeast Africa, with a population of 17 196 629 is one of the least developed and impoverished countries in the world (Central Intelligence Agency 2020). Of the population, 62.1% are 15 years of age and older and most can read and write (69.8% men; 55.2% women). The economy is predominately dependent on agriculture, with corn and tobacco being the staple crops, and 80% of the population living in rural areas are employed in agriculture. There are financial implications for PLWA, in an impoverished agrarian society, who need employment and money for sun protective clothing, topical sunscreen and transportation to dermatology clinics. Clinics provide not only free sunscreen lotions but also information on albinism, which is essential for parents of children with albinism (CWA). Epidemiological studies are needed on Malawian PLWA who have ocular disorders and are employed in the agricultural sector, regardless of the harmful effects of UV rays on the eyes (WHO 2003). Poverty, illiteracy and ignorance, problems found in many parts of Africa, contribute to stigmatisation of people with albinism (Cruz-Inigo et al. 2011). In communities steeped in traditional myths and beliefs, where knowledge of albinism is lacking, as in Malawi (Braathen & Ingstad 2006), PLWA are ostracised and sometimes kidnapped, mutilated and killed (Mwananyanda 2019).

In one research project, individual in-depth interviews with 25 Malawian PLWA and their family members focussed on knowledge, beliefs and behaviour related to albinism (Braathen & Ingstad 2006). Systematic reviews of OCA and sun-induced adverse health effects on the skin and eyes of individuals with OCA in sub-Saharan Africa excluded Malawi (Hong et al. 2006; Wright et al. 2012). No studies could be found that have focused on the Malawian context, specifically exploring the experiences, perceptions and understanding of albinism from the perspective of mothers of CWA, which is the focus of this study.

## Methodology

### Design of the study

‘A qualitative descriptive study was the method of choice because straight descriptions of phenomena were desired’ (Sandelowski 2000:334). This design draws on the tenets of naturalistic inquiry to study something as it is (Creswell & Miller 2000).

### Participants and setting

The study population consisted of mothers of CWA who attended the dermatology outpatient clinic at the Queen Elizabeth Central Hospital (QECH) in Blantyre, Malawi, a government institution for referrals from surrounding districts. The hospital is located along the main transport route, increasing accessibility for patients. One day a week, the dermatology clinic attends to PLWA of all ages who have skin-related conditions and dispenses free topical sunscreen. Those who also have ocular conditions are referred to the ophthalmology department. The nature of the study required responses from persons with experience of the study’s focus, and therefore, purposive sampling of participants was employed to ensure rich information to achieve the aim of the study (eds. Denzin & Lincoln 2000).

The HCPs at the clinic selected the participants according to the following criteria: birth mothers, with or without albinism, aged 18 years and older, of CWA up to 18 years of age, who attended the clinic. Participants could speak English or Chichewa fluently. An individual under the age of 18 is considered to be a child (UNICEF 1990).

### Data collection and analysis

A two-part interview guide was constructed (N.L.) in English from the existing literature and examined, by the second and third authors of this article (U.K., T.d.V.), for logical layout, adequacy of content and length. Part 1 questions dealt with the mothers’ experiences and perceptions of having a CWA. Part 2 questions explored the mothers’ understanding of albinism. The interview guide was translated from English to Chichewa independently by a carefully screened school teacher and nurse, using forward and backward translation (Tsang, Royse & Terkawi 2017). They did not have albinism. Individual face-to-face interviews between the interviewer (N.L.) and the mothers were conducted between 28 June and 12 July 2018.

Data collection continued until no new data emerged from the interviews (Malterud, Siersma & Guassora 2016). On the day of appointment, the interviewer (N.L.) would approach potential participants individually introducing herself and the study. Participants would give written informed consent for voluntary participation after reading the contents of the Participant Information Sheet or having it read to them. The information given covered: the purpose of the study, ethical clearance, time and setting needed for the interview (30 to 45 min), nature of the interview, measures to ensure confidentiality of data and anonymity, and freedom to discontinue participation without penalty or refusal of further healthcare treatment. Refreshments were provided during the interview. The offer of a separate facility close by for participants’ own child minders was only taken up by one participant. Pseudonyms of the participants’ choice are used.

There is no fixed sample size in qualitative research but the number of participants is determined when ‘information power’ (Malterud et al. 2016:1758) is achieved. In the present study, a sample of ten participants was adequate to reach data saturation. Interviews were conducted in Chichewa, audio recorded and transcribed, and transcriptions were forward translated into English and back-translated into Chichewa (N.L.) (Tsang et al. 2017).

The standardised open-ended interview seemed the most suitable as participants were all asked identical questions ensuring a consistent approach (Turner 2010). Although the wording of questions was extremely structured, the questions allowed open-ended responses (Gall, Gall & Borg 2003) permitting participants to volunteer as much detailed information as they were comfortable with. It also allowed the interviewer (N.L.) to ask probing questions as the need arose to obtain thick, rich data (Creswell et al. 2007). Weaknesses with open-ended interviewing techniques include the vast range of responses resulting in difficulty extracting similar themes from the interview transcripts and coding the data (Creswell et al. 2007). However, the deep immersion in the data analysis process reduces researcher biases (Gall et al. 2003).

Thematic data analysis involved preparing, coordinating and examining the data, identifying themes and interpreting results systematically after familiarisation with the data, yet allowing for an eclectic but reasonable approach to analysis (Clarke & Braun 2013; Lewis 2015; Sandelowski 2000). Interview transcriptions were read and reread many times before extracting similar themes by manually colour coding the data (N.L.). Files containing colour-coded English transcriptions, a table with significant statements, formulated meanings extracted from these statements, and initial themes were encrypted and sent electronically to the collaborating researchers (co-authors U.K. and T.d.V.) in South Africa. Each author independently examined the themes and suggested modifications; changes were accepted when consensus had been achieved. Participants were then consulted telephonically by prior arrangement (N.L.), to confirm the final themes (and to achieve rigour of the findings by member checking) (Trochim 2006); there was no disagreement.

At the completion of the study, two checklists were completed (N.L.) to achieve transparency in reporting the study and its findings and to allow replication: a 32-item checklist, the Consolidated Criteria for Reporting Qualitative Studies (COREQ) (Tong, Sainsbury & Craig 2007) and a 21-item checklist, the Standards for Reporting Qualitative Research (SRQR), to evaluate the quality and strength of the completed study (O’Brien et al. 2014). Information in both checklists was found to be satisfactory (U.K., T.d.V.) and an accurate record was maintained.

### Ethical considerations

The study was approved by the Human Research Ethics Committee (HREC) of the Faculty of Health Sciences of the University of Cape Town (HREC 828/2017) and the National Committee on Research in the Social Sciences and Humanities in Malawi (NCRSH P.05/18/271).

## Findings

Information power (Malterud et al. 2016) was achieved after interviewing 10 participants and their demographics are presented in Table 1.

**TABLE 1:** Demographic characteristics of study participants (*n* = 10 Malawian mothers of a biological child with albinism).

Participant pseudonym	Age in years	Mother has albinism	Marital status	Age of CWA (years or months)	Gender of CWA
Clara	35	No	Married	4 months	boy
Martha	29	No	Single	8	boy
Ida	32	No	Divorced	6	boy
Agi	29	No	Single	2	boy
Catherine	40	No	Married	4	girl
Doreen	43	Yes	Married	10	girl
Chrissy	30	No	Married	6	girl
Linesi	33	Yes	Married	9	boy
Fatima	26	No	Married	7	boy
Fanny	31	No	Divorced	7	boy

Note: Age and gender were reported in field notes taken during the interviews N.L.

CWA, child with albinism.

The mean age of the participants was 33 years. Two participants had albinism: Doreen, 43 years old, and Linesi, 33 years old. Both participants were still married, as were four others. The demographic characteristics of the CWA presented in Table 1 refer only to the children who attended the clinic on the day of the interview. Details of other siblings who may have had albinism were not requested but were referred to by some of the participants.

Themes that emerged from descriptions of mothers’ experiences related to the chronology of events (Sandelowski 2000): how they felt after seeing their baby for the first time; reactions of nurses or midwives and family members to them and their child; raising a CWA in their community; and their lack of knowledge and understanding of albinism.

### How participants felt after seeing their baby for the first time

Sub-themes emerged from participants’ descriptions of their reactions to seeing their baby for the first time: (1) they expressed degrees of ownership or distancing, for example, using words such as ‘my child’: Ida, Catherine, Clara; ‘the child’: Fanny, Linesi (who had albinism); ‘a child with albinism’: Chrissy; ‘children of this kind’: Martha, Fatima; and ‘it’: Agi, Doreen (who had albinism); (2) acceptance of their child, which was immediate for Ida, Catherine, Linesi, Chrissy, Clara and Agi *versus* expressions of initial pain (Doreen and Fanny) and disappointment (Martha), or being non-committal but anticipating skin problems in their child (Fatima). The role played by their religious beliefs with regards to the participants’ acceptance of their children was clearly described by Martha, Agi, Catherine, Doreen and Chrissy. In summary, responses towards their child with albinism ranged from immediate gratitude and happiness to initial emotional pain, disappointment and concern.

### Participants’ perceptions of reactions of nurses or midwives and family members towards them and their baby

Descriptions were of initial surprise shown by some HCPs and interpreted by Clara, Catherine and Linesi as a phenomenon not often encountered. But in one case, the surprise was ‘because his skin was clear with no black spots nor rashes …’ (Martha). Some HCPs expressed happiness, which Ida surmised was probably intended not to disappoint the mother, and one reportedly advised Agi to obtain supplies of topical sunscreen from the hospital. Both Chrissy and Fatima who had had a caesarean section did not remember the HCPs’ reactions.

Conversely, some HCPs’ lack of patient education and vagueness resulted in confusion and the mothers’ distrust of their competence:

‘Yes, there were Doctors who told me that my child had no problems and that I must take care of the child. I just do not understand why things happen that way’. (Fanny)

‘They … did not tell me anything. I was only told how to care for my child at a certain age at the Under-Five clinic. Because of ignorance maybe.’ (Doreen)

‘The midwives … said the child is a human being but different from other children.’ (Linesi)

One participant expressed a very real sense of fear and that harm was intended to her infant:

‘They did not say anything to me or show me my baby but went straight to my mother and asked what they should do with the baby … maybe some people ask midwives to kill their children when they are born with albinism’. (Catherine)

Acceptance of a CWA by family members was either unconditional (Ida, Agi) or conditional:

‘… my husband’s family did not accept the child … My husband sent me home to my parents for six months till they got back to their senses. … because my husband does not have albinism’. (Catherine)

Some paternal families made hurtful accusations (Catherine, Fanny and Chrissy) that the participants must have slept with a man with albinism to have given birth to a CWA:

‘My family accepted my child and they love him but my husband and his family did not accept the child and their relationship with the child is not good. I … heard that they think I had slept with another man with albinism because the father is black in complexion’. (Fanny)

Disappointment amongst relatives was linked to the participant being the first one in the family to have given birth to a CWA and to difficulties associated with caring for such children in protecting them from harmful effects of the sun (Clara).

Three participants experienced mockery by their respective families, and this was painful. Fatima attributed the mockery to lack of understanding, whereas Chrissy thought that her sister-in-law may not have seen a CWA before:

‘Some … mocked me … how could I give birth to a child who stinks or smells like the sun he is going to bring calamity to us. … because it was surprising and strange to them’. (Martha)

Surprisingly, Doreen, who had albinism, and two CWA reported that her family referred to her youngest child as ‘the white person’ and, whether in jest or not, said that they were going to sell her. Remarkably, Linesi, who also had albinism, made little contribution to this question.

### Raising a child with albinism in the community

Some sub-themes described above re-emerged from the data for this section. Themes included: beliefs and myths, social isolation and discrimination, mockery, the mothers’ fear of their children being abused and killed, divorce, financial deprivation and implications for educational opportunities.

#### Beliefs and myths

‘Pregnant women also chase him, saying he will bring calamity … will make them deliver a child with albinism’. (Martha)

‘… but other people spit and some parents tell their children to touch their hair when they see her. I think and I have heard that they do so to prevent their children from turning into a child with albinism’. (Catherine)

‘…they think a person with albinism is not a human being, does not live long, just disappears’. (Doreen)

Chrissy’s neighbours discouraged her from collecting sunscreen at the clinic, believing that the staff use this to entice PLWA for evil purposes, but Chrissy refuted this because during the 6 years she had attended the clinic no harm had come to her child.

#### Social isolation and discrimination

Martha, Chrissy, Linesi and Doreen felt despised by family and community members. Doreen’s 18-year-old daughter with albinism feared dating a ‘normal man’ out of distrust:

‘… his friends isolate him because of his appearance … some relatives and friends segregate the child sometimes …’ (Clara)

‘…He is segregated by friends at school to the point that he refuses to go to school sometimes … friends refuse to play with him’. (Martha)

#### Mockery and name calling

The participants experienced mockery of their children and name calling as particularly painful because it entrenched their own sense of social isolation and humiliation caused by others who discriminated against their CWA:

‘His friends mock him, isolate him and call him a white person….’ (Martha)

‘I have been despised that I and my children resemble a pig’. (Doreen)

‘Some relatives call her names such as napweri [dry pigeon peas], mzungu [white person]. People say that they stopped eating pork because of me, that I resemble a pig’. (Doreen)

#### Fear

Fanny was especially fearful at night and slept fitfully because a PLWA had told her that one night he had been stabbed whilst successfully warding off people trying to amputate his genitalia. Fatima expressed awareness from a supposedly reliable source that CWA are at risk of disappearing or being killed. She recalled a recent incident when she could not find her son outside the house and was told that he had followed a man who summoned him. Fatima and her neighbour ran after them, but the man suddenly disappeared and when asked, her son said that he had told him he is a ‘bwana’ [‘sir’], leaving Fatima even more fearful:

‘I feel worried especially when I hear stories of people with albinism being abused and killed, I feel sorry for myself and scared …’ (Clara)

‘People call my child makobiri [money]. They say my child can be a source of money if I sell her. Some friends have suggested to me that hospital staff might have exchanged my child with the one I have’. (Chrissy)

‘There are times when some people have said to me … why I did not kill the child when he was just born’. (Agi)

Agi’s report above was supported by Fanny who described how some of her friends have asked her directly why she does not kill her child. She dissociated herself from them.

#### Marital relationships

Catherine felt disgraced when her husband sent her to her parents for 6 months after the birth of her CWA. Another participant stated that relatives made particularly unkind comments:

‘The main challenge I have gone through is losing my marriage. My ex-husband’s relatives were talking because my husband does not have albinism so I decided to get out of the marriage and take care of my child’. (Ida)

#### Socio-economic and educational implications

The economic challenges the mothers’ face because of the stigmatisation associated with albinism are far reaching. To ensure her child’s safety, Catherine placed her in a private school close to home. Despite having a Malawi School Certificate of Education, Doreen’s 18-year old daughter with albinism struggled to find a job. Doreen could not afford to buy a hat or long-sleeved clothes and had no money for transport to the clinic to collect sunscreen for herself and her children. Chrissy suggested that an adequate supply of topical sunscreen should be provided between clinic appointments to avoid having to make extra trips to the clinic. Fatima, a first-time mother with financial problems had not been informed about the free supply of topical sunscreen at the QECH and was paying between 82 and 164 South African rand (ZAR) for this from a PLWA.

Fanny and Linesi disclosed that their children had skin and eye problems. Fanny’s parents supported her son financially because his father did not. To help him progress at school, she asked the teacher to write a little bigger on the chalkboard to enable him to read. Linesi could not afford spectacles but the teacher moved her daughter to the front of the class to make reading from the board easier.

#### Positive experiences

There were three accounts of CWA being totally accepted within their neighbourhoods (Ida, Fanny, Fatima), for example:

‘… they help me to take care of the child and advise me not to leave the child alone. One of my neighbours has a child with albinism and she … informed me that free sun cream is provided at the [*QECH*] dermatology clinic’. (Fatima)

### Mothers’ lack of knowledge and understanding of albinism

Themes emerged from participants’ descriptions of understanding of albinism related to the cause of albinism, information given by HCPs (doctors, nurses or counsellors) after the birth of their child, reasons for the white appearance of the skin and their poor eyesight, their source of information and the type of information on albinism, their understanding of and response to this information.

Participants had inadequate or erroneous information on the cause of albinism. Martha, Doreen and Chrissy reported that the Malawi Broadcasting Corporation radio series had denounced the practice of stigmatising PLWA but provided no information on the cause or management of the condition. Most of our participants did not report actively seeking information to empower themselves, but did not elaborate on this. Clara, for example, passively relied on others for information:

‘they did not say anything to me…; they did not tell me anything’. (Clara)

Six participants were evasive; although their children looked different, they were no different to other sentient human beings. Examples of some explanations:

‘… when genes are weak, a child fails to develop the top layer skin … I was given an example that if a normal person has an open wound, a white skin appears first before blood comes out’. (Ida)

‘… paint in the abdomen [*of men*] but I have forgotten its name … I don’t know what happens but, it can cause albinism’. (Chrissy)

‘… people say it is caused by “*mwanamphepo*” [an inborn illness]’. (Fatima)

Six participants did not remember hospital staff giving them information about albinism. The remaining participants only recalled information given about the need for skin protection:

‘I was told that this child is different from any other child and needs to be protected from sun and they referred me to this hospital where I get a supply of sun burn cream’. (Clara)

‘I was told that he should put on long-sleeved clothes, sun hat and I should apply sun burn cream from the hospital’. (Agi)

‘I delivered through Caesarean section … I was told nothing by the medical people, they just sent me to cotton weaving company where I got information on how to care for my child’. (Chrissy)

‘I was not told why I gave birth to this kind of a child’. (Fatima)

The data generated from this small study suggest that the participants have little understanding of albinism. If HCPs had adequate knowledge of the genetics and inheritance of albinism, considering that PLWA are treated at a special dermatology clinic, this knowledge was not evident from the participants’ explanations.

## Discussion

Lack of knowledge and understanding of albinism by society, but surprisingly also by PLWA, is well documented (Baker et al. 2010; Cruz-Inigo et al. 2011; Phatoli, Bila & Ross 2015). This is also the case in Malawi (Braathen & Ingstad 2006). Our study findings, which aimed to describe mothers’ experiences, perceptions and understanding of having CWA in Malawi, support the published data. Lack of knowledge is at the core of the stigma and prejudice (Pryor & Reeder 2011) associated with albinism and its consequences.

### Lack of knowledge and understanding of albinism

Mothers who give birth to children with genetic conditions should receive information and counselling from HCPs (Baker et al. 2010; Rantanen et al. 2008) but Malawian mothers reportedly had no understanding of the cause of albinism in their children (Braathen & Ingstad 2006). Two participants in our study had albinism, one of whom was the oldest and had given birth to two CWA. Surprisingly, she had very little knowledge and understanding of the condition. Knowledge of the cause of albinism, before leaving the hospital with newborn children, empowers mothers to disabuse family members and the community of misunderstandings they may have about the condition, which may result in non-acceptance of the mother and/or the child when they go home (Cruz-Inigo et al. 2011). In Venda, South Africa, a woman who was ashamed of her baby with albinism hid her until she had received genetic counselling after which she ‘… was able to take her child out because she was able to explain the genetic cause of the condition’ (Baker et al. 2010:171). Nurses should be familiar with the genetic disorders seen in their communities, have knowledge of referral systems and be prepared to discuss how these conditions are inherited.

All 10 CWA in our study had OCA, with visual impairment, and had inherited a mutated gene from both parents. As far as the participants could remember, HCPs gave them no information on the cause of albinism after the birth of their babies. Had the three participants who were reportedly falsely accused of infidelity by their husbands, families and communities known about genetic inheritance in albinism, they would have had the knowledge and possibly the confidence to defend themselves. Instead, lack of information resulted in feelings of shame, anger and emotional trauma in these mothers. Some participants were given instructions by HCPs on the need for skin protection, as in a previous Malawian study about knowledge and beliefs from an African setting (Braathen & Ingstad 2006).

In our study participants described the causes of albinism as ‘paint found in men’s abdomen’, ‘problems found in man and woman’ and, as described by Tolhurst et al. (2008:89), ‘*mwanamphepo’* [an inborn illness]. Phatoli et al. (2015) found that three of 10 participants without albinism investigated in a South African student sample had no idea what albinism was, five had some information gleaned from the media but without fully understanding the cause of the condition, whilst two demonstrated knowledge of the condition. The level of understanding of albinism by the five PLWA in their study was interpreted as being based on their personal experiences of having the condition, on how it affected their lives and their awareness of the stereotypes and beliefs around albinism (Phatoli et al. 2015). Of the five students, three understood the medical (hereditary) aspects of albinism. When extrapolated to our study, the difference is that the CWA were not interviewed. Therefore, the mothers’ self-reported lack of knowledge could possibly be attributed to not having the condition and being personally removed from it to some extent, but it raises questions about the mothers who themselves had albinism.

A survey conducted in Zimbabwe found that many CWA did not know the cause of albinism. Instead, they cited stories implicating witchcraft and God and they associated albinism with punishment and mockery (Cruz-Inigo et al. 2011). However, lack of awareness is not restricted to the public, as HCPs often also lack understanding of albinism because of its relative infrequency (Baker et al. 2010). This may account for the midwives’ shocked reactions reported by two of our participants when they saw their babies.

Our participants reported that the Malawi Broadcasting Corporation radio series denounced the practice of stigmatising PLWA but had provided no further helpful information. Genetic nurses in northern South Africa reportedly give radio talks on albinism (Cruz-Inigo et al. 2011). As patient engagement and education is core to nursing practice, information about albinism from nurses would presumably be more easily understood by the population than presentations by reporters. Surprisingly, most of our participants did not report actively seeking information to empower themselves. This could be because of the unavailability of other sources of information about albinism as the only media mentioned by participants was the radio. The media have an important role to play in educating the public on inheritance of albinism, but instead have been awash with sensational stories of abductions and killings (Mwiba 2018) which may entrench stigmatisation.

### Stigmatisation and its consequences

In the context of PLWA and particularly CWA, stigmatisation has far reaching effects. Cruz-Inigo et al. (2011) found that poverty and illiteracy in many parts of Africa may result in lack of awareness of the causes of albinism which contributes to stigma. Researchers stand accused of not communicating study results adequately to society, resulting in misunderstandings and the formation of a stigma cycle (Baker et al. 2010). One consequence of stigmatisation is social isolation and discrimination.

Because of male dominance in African countries, women are blamed for producing a child with albinism. As in our study, Wan (2003) reported incidents of wrongful accusations of infidelity of some women who give birth to CWA, thereby dishonouring their intimate relationships. Others may be cursed and their husbands may divorce them (Cruz-Inigo et al. 2011; Franklin et al. 2018; Masanjala, Mvena & Kayunze 2014). Six of the participants in our study were still married at the time of the study, and four had been abandoned by their husbands because no one in the husband’s family had albinism.

A study conducted in Malawi reported that the very visible difference in appearance of PLWA can lead to lack of acceptance and a poor social life (Lynch, Lund & Massah 2014). Baker et al. (2010) reported the case of a woman with albinism in Zimbabwe who, despite having the proper qualifications, was not reportedly employed as she was considered unattractive. A study conducted in Tanzania showed that PLWA are considered disabled and marginalised (Masanjala et al. 2014) and may be labelled as ‘other’ as a consequence of societal beliefs associated with albinism. Such beliefs may be promoted by people in the community to justify protecting themselves from death, curses, suffering and evil (Imafidon 2017). ‘The beliefs surrounding albinism in southern Africa are often found to compensate for such a lack of knowledge’ (Baker et al. 2010:170). In our study, four participants were shunned by their friends and felt isolated because of their children’s appearance. One child refused to go to school; such is the power of stigmatisation to affect academic progress, mental health and professional achievement (Watson 2012).

To be accepted fulfils a basic human need (Chang & Schaller 2000). Our study confirmed findings from the Braathen and Ingstad’s (2006) Malawian study of mothers of CWA who expressed love for their children, believing they are a gift from God. Five participants used religion (God) as the reason for their acceptance of their child. Brocco (2015) reported that many parents believed that albinism is the will of God, who is the primary source of creation and should not be questioned, which may account for none of the participants in our study expressing parental guilt. Three of the mothers in our study (who did not have albinism) expressed immediate love and acceptance of their CWA after their birth, using words of ownership (‘my child’) which implied a sense of acceptance and relationship. For three mothers, including one with albinism, acceptance seemed somewhat delayed once they had come to terms with their self-reported disappointment and pain of having given birth to a CWA because of the implications for themselves and their children. Surprisingly, two of the mothers, one of whom herself had albinism, referred to their children as ‘it’. Bos et al. (2013) described internalised stigma as a feeling of loss of self-esteem, accompanied by psychological distress as a result of discrimination by society, which might account for these mothers apparent distancing of themselves from their CWA. The concept ‘maternal subjectivity’ best describes the mothers’ responses as ‘founded on both being for the self and for another – one’s child – and thus as something that is paradoxically connected and separate’ (Harvey 2015:93). In a South African metropolitan study (Kromberg 1987), significantly more mothers of CWA than controls reported postnatal headaches, feelings of depression and not wanting to hold or breast feed their babies, even making plans to return to their families in rural areas. Reasons cited were complaints about the infants’ hair and skin colour.

Acceptance builds self-esteem and psychosocial well-being (Kromberg 2018b). In our study, there were three accounts of CWA being totally accepted within their neighbourhoods. Such acceptance is also reported by Braathen and Ingstad (2006). To be accepted by a black African community, when one has the facial features of that community but not the same inherited skin or hair colour, is challenging. One of the mothers with albinism (Doreen) had an adolescent daughter who will need parental support to achieve her full potential because, despite having the required qualifications, she was unable to find employment because of her appearance. Themes that emerged from a qualitative study investigating the perceptions of 12 adolescents with visual impairments on the social support they received from their parents illustrated processes related to emotional, informational and tangible support (Chang & Schaller 2000). Doreen did not display any knowledge of the aetiology of albinism so her ability to provide informational support to her adolescent daughter is questionable.

Visual impairment associated with albinism is a potential impediment to socioeconomic advancement if no provision is made for efficient management of eye healthcare services. If there is ineffective intervention for visual impairment associated with albinism at a national level, targeted universal eye health objectives of the Vision 2020: The Right to Sight project will not be achieved. The objectives are: (1) to eliminate causes of avoidable blindness, (2) the development of human resources and (3) the provision of appropriate technology and infrastructure (Holland & Resnikoff 2019; Resnikoff 2000). If CWA are not educated, discrimination continues into adult life because they may be unemployable, in this way violating the following United Nation’s Sustainable Development Goals: No. 3 (good health and well-being), No. 4 (quality education), No. 8 (decent work and economic work) and No. 10 (reduced inequalities) (United Nations 2019). Educational institutions are an integral part of the formal social support system and should provide students who have visual impairments with special support to meet their educational needs in mainstream education in Malawi and to ensure their efficient functioning within society (Lynch et al. 2014). Low vision aids are advocated for PLWA who have more serious eye conditions than refractive errors that can be corrected with spectacles (Minto & Awan 2004).

CWA are judged by their appearance, but when given the opportunity to realise their potential, they can succeed in life (Baker et al. 2010). George and Duquette (2006) described a case study of a Grade 6 student with albinism who progressed academically in a rural school in Ontario, Canada, largely because of his mother’s support and the chance to explore his potential. In our study, one participant intervened on behalf of her son who could not see the teacher’s writing on the board. Teachers should be trained to recognise poor vision, as reported by one participant in our study, and in basic eye testing techniques, particularly in CWA, in situations where school health nurses are not available. Inadequate knowledge and understanding of albinism may result in mockery and even harm to CWA, thereby depriving them of opportunities to develop their potential.

Myths, resulting from lack of awareness and a misunderstanding of albinism in Malawi and many other African countries, as found in our study, have led to PLWA being mocked. Name-calling such as ‘mizukwa’ (ghosts), ‘napweri’ (tomato with white spots) and ‘mzungu osauka’ (poor white person) is painful and results in social isolation and humiliation (Baker et al. 2010; Cruz-Inigo et al. 2011; Imafidon 2017; Mwiba 2018). Two participants in our study who had albinism alluded to the myth about ghosts: in Zimbabwe, people with albinism have been considered to be water spirits (Machoko 2013). Baker et al. (2010:174) described the fear of contagion associated with albinism.

CWA, in particular, have been attacked, kidnapped, mutilated and/or killed by people practising witchcraft or for the sale of body parts (Cruz-Inigo et al. 2011; Mwiba 2018). Two participants in our study were asked by community members why they allowed their children to live and one participant reported living in fear, especially at night after a man with albinism had been stabbed by people attempting to amputate his genitalia. Interventions by the Malawian government to protect the rights of PLWA include a national disability policy, which was finalised in 2005 (Braathen & Ingstad 2006). In Tanzania, such measures include the provision of mobile telephones, the nomination of a woman with albinism to parliament and burning witch doctors’ licences (Kisanga & Mbonile 2017). The high level of illiteracy in Tanzanian communities, compounded by the low level of secondary and tertiary education, resulted in discrimination that perpetuated the disregard of human rights of PLWA and unequal access to education (Kisanga & Mbonile 2017). The high level of illiteracy in Malawi and Tanzania may contribute to the reasons for the failure of these interventions in these countries but this has not been reported. For these reasons, a special boarding school was opened in Tanzania to protect CWA who had been abused and abducted (Brocco 2015). Although no published evidence was found to support our participants’ perceptions that registered nurses and midwives in labour wards in Malawi may be involved in killing newborns who have albinism, there are accounts of traditional midwives committing infanticide in instances of albinism and then reporting a stillbirth (Cruz-Inigo et al. 2011).

Journalists have risked their lives to report the occult-based killings of PLWA (in parts of Burundi and Tanzania) for use as talismans to bring luck and wealth: a complete set of body parts; four limbs, genitals, ears, tongue and nose may earn the equivalent of 75 000 US dollars (International Federation of Red Cross and Red Crescent Societies 2009). In February 2019, Amnesty International reported an escalation in the number of killings and other human rights abuses, including abductions and robberies against PLWA in Malawi since November 2014 (Mwananyanda 2019). Mwananyanda (2019) estimated the population of PLWA in Malawi to be between 7000 and 10 000, representing a ratio of 1 in every 1 800 persons and reported that two fatalities and three abductions had been reported since December 2018, two of whom were rescued by community members. Living with constant fear is an infringement of the right to life of this population and does not only have psychosocial effects for the person involved (Hong et al. 2006) but also for mothers of CWA (Kisanga & Mbonile 2017). Torner, a Tanzanian advocate for PLWA, made a plea to local and international organisations to assist in protecting the rights of PLWA as human beings (Allawh & Norton 2014).

Mothers of CWA face many challenges. Lynch et al. (2014) recommended that governments should support parents in decision-making regarding their children’s education and future employment prospects. More research and funding are needed for awareness campaigns and workshops and for the establishment of counselling centres in health settings where mothers of CWA can be educated about the condition (Cruz-Inigo et al. 2011; Imafidon 2017).

## Limitations of the study

A pilot study was not conducted to test the interview guide for flaws or other weaknesses and to allow for revision (Brinkmann & Kvale 2018; Turner 2010). This omission resulted in little variation in participants’ responses to two questions. These questions could have been merged and more questions added. Of more importance, a pilot study may also have shown the limitations of the research design. ‘Researchers conducting qualitative descriptive studies stay close to their data and to the surface of words and events’ (Sandelowski 2000:334). Fidelity to this approach resulted in superficial descriptions and therefore ‘thin’ rather than thick, rich data (Creswell et al. 2007). Descriptions provided by two participants who had albinism, one of whom had two CWA, were particularly disappointing and lacking in substance. Employment of a phenomenological design may have been a better match for a sensitive topic such as this study of mothers’ experiences of raising CWA. As none of the researchers have albinism or CWA in this study, the pain experienced by the mothers, particularly those who themselves had albinism, could not be underestimated. Also, the participants’ fear of full disclosure to a stranger might not have been fully understood, and this may account for their perceived reticence.

Backward and forward translation (Tsang et al. 2017) of the interview guide, participant information sheet and consent form and of the transcriptions between English and Chichewa was fraught with logistical difficulties such as finding qualified translators. Selection of participants by HCPs at the dermatology clinic not familiar with qualitative research might have led to the exclusion of participants who were more able to provide thick, rich data (Creswell et al. 2007) than those who were interviewed. Limiting the collection of data to 3 weeks and including only mothers and not HCPs or community members might have limited the scope, richness and usefulness of the study data. Telephonic rather than face-to-face discussions for member checking may have limited dependability and credibility of the analysed data for emergence of themes. Further, as the sample in this study was not a representative one, as it was small and collected from a hospital-based clinic, results cannot be generalised to the larger population of mothers with CWA and PLWA living in Malawi.

## Conclusion

A description of perceptions, experiences and understanding of a small sample of mothers of children living with albinism in Malawi has revealed that they and their children are stigmatised and may be unsafe in their communities. Nevertheless, these mothers were positive about accepting and loving their children and attempted to protect them from harm whatever the cost. Overall, the mothers’ understanding of albinism was poor as they had reportedly been given inadequate information by HCPs. These findings should lead to increased awareness and the provision of improved counselling by nurses and other HCPs. To our knowledge, this is the first reported Malawian study of mothers’ perceptions and experiences; it has begun to address a gap in the existing knowledge in this field and provides a foundation for further research, specific to the larger population of mothers with CWA and PLWA in Malawi.
